# Genetic Testing Abnormalities in Children with Autism Spectrum Disorder: Prevalence, Patterns, and Clinical Associations

**DOI:** 10.3390/neurolint18090172

**Published:** 2026-09-12

**Authors:** Viswabhaskar Susarla, Mariah George, Ananthi Rathinam, Danish Bhatti

**Affiliations:** 1 Kids Neuro Care LLC, 10931 Dylan Loren Cir, Orlando, FL 32825, USA; 2 College of Medicine, University of Central Florida, 6850 Lake Nona Blvd., Orlando, FL 32827, USA; ma030549@ucf.edu (M.G.);

**Keywords:** autism spectrum disorder, genetic testing, genetic findings, copy-number variation, seizures, electroencephalography, magnetic resonance imaging, neurogenetics, retrospective cohort

## Abstract

Background: Genetic testing in autism spectrum disorder (ASD) can reveal a wide range of chromosomal and sequence-level abnormalities, yet large real-world neurology cohorts rarely report the full spectrum of findings alongside clinical correlates and patterns of testing. We characterized genetic findings in an 1884-patient ASD cohort and examined factors associated with both abnormal findings and the use of genetic testing. Methods: This retrospective cross-sectional study included 1884 patients. Genetic testing was performed in 1011 patients; the primary outcome was an abnormal versus normal genetic result among those tested. All 371 abnormal findings were catalogued in a de-identified supplement. The primary multivariable model included age, sex, seizure history, and EEG abnormality (N = 901); an MRI-inclusive sensitivity model used 422 complete cases, and a separate full-cohort model evaluated factors associated with genetic testing. Results: Abnormal genetic findings were documented in 371/1011 tested patients (36.7%, 95% CI 33.7–39.8%). In the primary model, female sex (aOR 1.483, 95% CI 1.089–2.018; *p* = 0.012) and seizure history (aOR 1.634, 95% CI 1.136–2.352; *p* = 0.008) were independently associated with abnormal findings, whereas EEG abnormality was not significant after adjustment (aOR 1.373, *p* = 0.084). In the MRI-inclusive sensitivity model, female sex and seizure history remained significant, while MRI abnormality was not independently associated (aOR 1.235, *p* = 0.302). Genetic testing was more common among patients who also underwent EEG or MRI (both *p* < 0.001). Conclusions: More than one-third of tested patients had an abnormal genetic finding, spanning a broad range of copy-number, sequence, homozygosity, Fragile X-related, chromosomal, and syndromic findings. Seizure history and female sex were the principal adjusted correlates of abnormal findings. Genetic testing clustered with EEG and MRI use, reflecting observed patterns of neurological work-up within this cohort rather than temporal or causal relationships.

## 1. Introduction

Autism spectrum disorder (ASD) is a heterogeneous neurodevelopmental condition with substantial genetic contribution to liability [1,2,3,4]. Recent U.S. surveillance estimates ASD prevalence at approximately 1 in 31 children aged 8 years, underscoring the scale of etiologic evaluation encountered in clinical practice [2]. At the molecular level, ASD risk spans common variation, copy-number variation, and rare sequence variation affecting pathways that include synaptic function, transcription, and chromatin regulation [5,6,7,8,9,10].

Professional guidance supports etiological genetic evaluation in ASD and related neurodevelopmental disorders. Chromosomal microarray has long served as a first-tier approach for developmental disability and congenital anomalies, and exome or genome sequencing is increasingly recommended early in the evaluation of neurodevelopmental disorders; the American Academy of Pediatrics also incorporates genetic testing into the medical evaluation of ASD [11,12,13,14,15]. Published yields vary substantially across assays and referral populations, emphasizing that the clinical meaning of a genetic result depends on both the testing modality and phenotype [16,17,18].

Real-world clinical records introduce a different challenge: testing modalities, report formats, and the level of retained laboratory detail are often heterogeneous. For a large chart-based cohort, forcing every historical finding into a contemporary reinterpretation framework can create additional misclassification when the original evidence is incomplete. We therefore analyzed abnormal genetic findings as documented in the clinical record and separately catalogued the available finding descriptions. Contemporary sequence-variant and copy-number interpretation standards provide important context [19,20], but this study was designed as a clinical-record cohort rather than a retrospective variant-reclassification study.

Genetic findings also intersect with the neurological phenotype. Genetically defined ASD cohorts show enrichment for epilepsy and other neurological complexity, and contemporary neurodevelopmental genetics cohorts suggest that diagnostic yield can vary by syndromic features and sex [21,22]. Epilepsy is itself common in ASD, and epileptiform EEG abnormalities can occur with or without recognized clinical seizures [23,24,25]. These relationships motivate multimodal analysis but do not establish temporal or causal direction in a cross-sectional record.

A second challenge is selective testing. In routine care, genetic testing is not performed uniformly; testing may vary with age, phenotype, prior investigations, family factors, and practice patterns. Correlates of an abnormal finding among tested patients should therefore be distinguished from correlates of undergoing testing. In this 1884-patient specialty-practice ASD cohort, we aimed to (1) quantify abnormal genetic findings among tested patients; (2) characterize the full spectrum of documented abnormalities; (3) evaluate clinical and neurodiagnostic correlates of abnormal result status; and (4) identify factors associated with genetic testing in the full cohort.

## 2. Materials and Methods

### 2.1. Study Design and Setting

**Figure 1:**
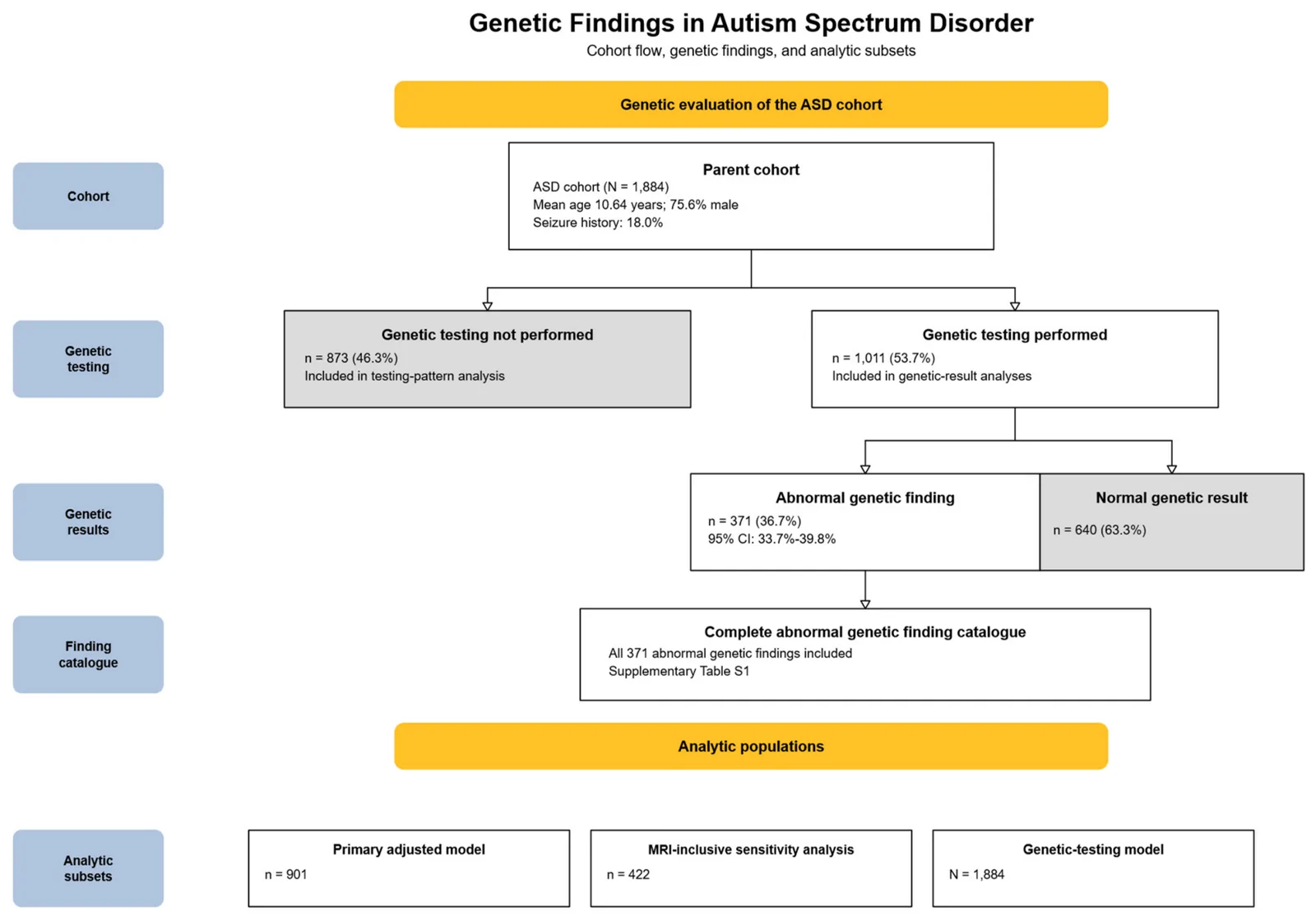
Cohort flow, genetic testing, abnormal genetic findings, and analytic subsets. CI = confidence interval; MRI = magnetic resonance imaging.

This study was a retrospective cross-sectional analysis of a defined clinical cohort evaluated at Kids Neuro Care LLC, a large-volume, multidisciplinary neurology practice. The parent analytic cohort contained 1884 patients with a documented ASD diagnosis and available demographic information. Although the practice is pediatric, 170 patients (9.0%) were aged 18 years or older at the recorded evaluation; “patients” is therefore used throughout. The analytic extract did not retain visit or test dates, so the temporal order of genetic testing, EEG, MRI, and seizure documentation was not considered (Figure 1).

Reporting was developed with reference to the Strengthening the Reporting of Observational Studies in Epidemiology (STROBE) statement [26].

### 2.2. Cohort and Variables

The parent cohort comprised 1884 unique patient records after dataset cleaning. Age was analyzed continuously in years and, in an exploratory analysis, categorized as 0–4, 5–9, 10–14, 15–17, and ≥18 years. Sex was recorded as male or female. Seizure history was considered present when a clinical or caregiver-reported seizure history was documented; detailed seizure classification was not used for the present genetic-outcome analysis.

### 2.3. Genetic Testing and Primary Outcome

Genetic status was categorized as normal, abnormal, or not tested. Genetic testing was considered performed when either a normal or abnormal result was documented. The 873 patients in the not-tested category constituted the comparison group for analyses of testing patterns.

The primary descriptive outcome was an abnormal genetic or chromosomal finding among patients who underwent genetic testing. Result-specific analyses compared abnormal versus normal findings; patients who were not genetically tested were excluded from these analyses and were never treated as having a normal result.

### 2.4. Genetic Finding Spectrum and Supplement

For the 371 abnormal results, available free-text finding descriptions were reviewed to characterize the broad spectrum of abnormalities. Overlapping descriptive groups included gene or sequence variants, deletions, duplications, and regions of homozygosity or long runs of homozygosity; named syndromes or conditions were also captured when explicitly documented. Individual findings were not independently reinterpreted or reassigned to a different clinical classification [19,20].

All 371 abnormal findings are represented in Supplementary Table S1 using study-specific record numbers without original patient identifiers or demographic linkages. Finding detail was available for 368 of 371 abnormal results; the remaining three records were retained in the supplement without a narrative description. Assay-specific yields were not calculated because genetic-testing modality could not be uniformly assessed across the cohort.

### 2.5. EEG and MRI Variables

EEG and brain MRI status were each classified as normal, abnormal, or not tested. Binary EEG-abnormal and MRI-abnormal variables were defined only among patients who underwent the corresponding investigation; untested patients remained missing in result-specific analyses. Separate binary testing variables identified whether EEG or MRI had been performed for the full-cohort genetic-testing model.

### 2.6. Statistical Analysis and Outcome Hierarchy

Continuous variables were summarized using means and standard deviations. Age comparisons used Welch independent-samples *t* tests. Categorical comparisons used Pearson chi-square tests; Fisher exact tests were retained as robustness checks for 2 × 2 comparisons where appropriate. Effect sizes are presented as odds ratios (ORs) with 95% confidence intervals (CIs), and Cramer’s V is reported for selected contingency tables. Two-sided *p* < 0.05 defined statistical significance; no multiplicity adjustment was applied, so secondary and exploratory findings are interpreted cautiously.

The analytic hierarchy was centred on the genetics-specific primary model. The primary adjusted model included age, sex, seizure history, and EEG abnormality among 901 patients with both genetic and EEG results. An MRI-inclusive complete-case sensitivity model added MRI abnormality and included 422 patients with complete genetic, EEG, and MRI data. A separate full-cohort model (N = 1884) examined factors associated with genetic testing as a function of age, sex, seizure history, EEG testing, and MRI testing. Because test dates and indications were unavailable, the testing model is interpreted as an association with patterns of clinical work-up rather than a causal referral model.

Logistic models report likelihood-ratio chi-square tests, McFadden and Nagelkerke pseudo-R^2^, and the in-sample area under the receiver operating characteristic curve (AUC). Variance inflation factors assessed multicollinearity. The regressions are association models rather than validated clinical prediction tools. Analyses were performed in jamovi version 2.7 [27] using the R statistical environment [28].

## 3. Results

### 3.1. Cohort and Genetic Testing

The cohort included 1884 patients with a mean age of 10.64 years (SD 4.81; range 1.75–28.0 years); 1424 (75.6%) were male, 460 (24.4%) were female, and 339 (18.0%) had a seizure history. Genetic testing was performed in 1011 patients (53.7%), while 873 (46.3%) were not tested. Tested patients were younger than untested patients (10.34 vs. 10.99 years; Welch t = 2.91, df = 1814, *p* = 0.004). Sex and seizure history were not significantly associated with testing in unadjusted comparisons, whereas EEG and MRI status distributions differed strongly between tested and untested patients (both *p* < 0.001; Table 1).

### 3.2. Frequency of Abnormal Genetic Findings

Among the 1011 patients who underwent genetic testing, 371 had an abnormal result and 640 had a normal result. The overall abnormal-finding rate was therefore 36.7% (371/1011; exact 95% CI 33.7–39.8%). Because the cohort included heterogeneous genetic-testing modalities, this value is best interpreted as the overall frequency of documented abnormal findings among tested patients rather than as an assay-specific diagnostic yield.

### 3.3. Spectrum of Abnormal Genetic Findings

Finding detail was available for 368 of 371 abnormal results (99.2%). Among these 368 records, 127 (34.5%) described a gene or sequence variant, 77 (20.9%) a deletion, 77 (20.9%) a duplication, and 61 (16.6%) a region of homozygosity or long run of homozygosity; categories were overlapping because individual patients could have more than one type of finding. The overall spectrum ranged from copy-number changes and gene variants to chromosomal abnormalities, Fragile X-related findings, and recognizable neurogenetic syndromes.

A named syndrome or condition was documented in 65 of 371 abnormal-result records (17.5%). More frequently recurring labels included 16p12 deletion (n = 7), 15q deletion/CNV (n = 5), sex-chromosome aneuploidy (n = 4), Charcot–Marie–Tooth disease (n = 4), Sotos syndrome (n = 3), neurofibromatosis type 1 (n = 3), DiGeorge/22q11 deletion (n = 3), and 16p13.11 CNV (n = 3). Fragile X-related annotations were present in 27 records and included affected, premutation/intermediate/grey-zone, carrier, and other documented findings; some co-occurred with another abnormal genetic finding (Table 2) The complete deidentified catalogue of all 371 abnormal findings is provided in Supplementary Table S1.

### 3.4. Unadjusted Correlates of Abnormal Result Status

Among patients with a documented genetic result, seizure history was more frequent in the abnormal group than the normal group (82/371 [22.1%] vs. 90/640 [14.1%]; OR 1.73, 95% CI 1.24–2.42; *p* = 0.001). Among the 901 patients with both genetic and EEG results, an abnormal EEG was associated with abnormal genetic status in unadjusted analysis (79/170 [46.5%] vs. 252/731 [34.5%]; OR 1.65, 95% CI 1.18–2.31; *p* = 0.003). Among the 464 patients with both genetic and MRI results, abnormal MRI status showed a weaker, non-significant association (88/198 [44.4%] vs. 97/266 [36.5%]; OR 1.39, 95% CI 0.958–2.03; *p* = 0.083). Age (*p* = 0.161) and sex (*p* = 0.101) were not significant in unadjusted comparisons (Table 3).

### 3.5. Multivariable Associations with Abnormal Genetic Findings

In the primary adjusted model (N = 901), female sex (aOR 1.483, 95% CI 1.089–2.018; *p* = 0.012) and seizure history (aOR 1.634, 95% CI 1.136–2.352; *p* = 0.008) were independently associated with abnormal genetic findings. EEG abnormality was associated in unadjusted analysis but was attenuated after adjustment (aOR 1.373, 95% CI 0.958–1.969; *p* = 0.084), and age was not independently associated (aOR 1.017 per year, 95% CI 0.987–1.048; *p* = 0.270). The model was significant overall (likelihood-ratio χ^2^ = 22.2, df = 4, *p* < 0.001), with McFadden R^2^ = 0.0188, Nagelkerke R^2^ = 0.0333, and AUC = 0.590. Variance inflation factors ranged from 1.01 to 1.13, indicating no meaningful multicollinearity.

In the MRI-inclusive complete-case sensitivity model (N = 422), the associations with female sex (aOR 1.646, 95% CI 1.039–2.609; *p* = 0.034) and seizure history (aOR 1.654, 95% CI 1.041–2.626; *p* = 0.033) remained statistically significant. Neither EEG abnormality (aOR 1.417, 95% CI 0.902–2.224; *p* = 0.130) nor MRI abnormality (aOR 1.235, 95% CI 0.827–1.845; *p* = 0.302) was independently associated, and age remained non-significant. The sensitivity model was significant overall (χ^2^ = 16.7, df = 5, *p* = 0.005) with AUC = 0.608.

Both models therefore identified seizure history and female sex as the principal adjusted correlates of abnormal genetic findings, while EEG and MRI abnormalities did not add independent associations after covariate adjustment. The low AUC values indicate that these variables should be interpreted as cohort-level associations rather than as a clinical prediction tool (Table 4).

### 3.6. Factors Associated with Genetic Testing

In the full cohort, genetic testing was non-randomly distributed (Table 5). Each additional year of age was associated with slightly lower adjusted odds of genetic testing (aOR 0.976, 95% CI 0.956–0.995; *p* = 0.014), and seizure history was also associated with lower adjusted odds (aOR 0.683, 95% CI 0.531–0.879; *p* = 0.003). In contrast, patients who underwent EEG had higher odds of genetic testing (aOR 2.424, 95% CI 1.872–3.139; *p* < 0.001), as did patients who underwent MRI (aOR 2.245, 95% CI 1.835–2.746; *p* < 0.001); sex was not significant. The model had an AUC of 0.649. Because chronology was unavailable, these associations are best interpreted as the clustering of investigations within the clinical work-up rather than as causal ordering decisions.

### 3.7. Exploratory Age-Group Pattern

The age-group distribution differed by abnormal versus normal result status (Pearson χ^2^ = 12.7, df = 4, *p* = 0.013; Cramer’s V = 0.112). Abnormal-result proportions were 36.4% at age 0–4 years, 32.2% at 5–9 years, 43.6% at 10–14 years, 42.5% at 15–17 years, and 30.1% at ≥18 years. Adjusted standardized residuals showed relative underrepresentation of abnormal findings at 5–9 years (−2.587) and overrepresentation at 10–14 years (+2.922). Because continuous age was not significant in any adjusted abnormal-result model, this is treated as an exploratory non-monotonic pattern rather than evidence of a developmental age gradient.

## 4. Discussion

### 4.1. Principal Findings

The principal findings are threefold. First, genetic testing was performed in 53.7% of the 1884-patient ASD cohort, and 36.7% of tested patients had an abnormal genetic finding. Second, the abnormal findings were highly heterogeneous: the full catalogue includes gene and sequence variants, deletions, duplications, regions of homozygosity, Fragile X-related findings, chromosomal abnormalities, and named neurogenetic syndromes. Third, seizure history and female sex were independently associated with abnormal findings in the primary model and remained significant in the MRI-inclusive sensitivity analysis. EEG abnormality was associated only before adjustment, and MRI abnormality was not independently associated. Genetic testing itself clustered strongly with the use of EEG and MRI, reflecting observed patterns of neurologic work-up within this cohort. Because the analytic extract did not retain test dates or chronology, these findings should be interpreted as cross-sectional correlates rather than as temporal, causal, or predictive relationships.

### 4.2. Breadth and Heterogeneity of Genetic Findings

The spectrum of findings is a central strength of this study. A single abnormal-versus-normal endpoint conceals substantial biological diversity, whereas the catalogue demonstrates that real-world ASD genetic evaluation can identify copy-number changes, sequence variants, homozygosity patterns, chromosomal abnormalities, Fragile X-related findings, and recognizable syndromic diagnoses within the same clinical population. Published diagnostic yields vary substantially by assay and referral setting [16,17,18]; our 36.7% value integrates heterogeneous genetic testing and therefore should be interpreted as an overall abnormal-finding rate among tested patients rather than directly compared with any single assay-specific yield.

Providing the complete de-identified catalogue of all 371 abnormal findings allows us to inspect this heterogeneity directly and preserves rare findings that would disappear in summary categories. Contemporary interpretation standards remain relevant [19,20], but individual entries were not retrospectively reclassified beyond the documentation available in the clinical record.

### 4.3. Seizure History Was a Consistent Correlate

Seizure history was associated with approximately 1.6-fold higher adjusted odds of an abnormal genetic finding in the primary model and the MRI-inclusive sensitivity analysis. This is biologically and clinically plausible because many monogenic and syndromic causes of ASD include epilepsy phenotypes. A recent genetically defined ASD cohort reported epilepsy in 35.4% of genetically defined versus 16.4% of genetically undefined patients [21], while meta-analytic work has long documented the substantial co-occurrence of epilepsy and ASD [23,24]. Our data do not establish directionality: genetic abnormalities may contribute to seizure susceptibility, seizures may increase the likelihood or breadth of genetic evaluation, or both may reflect greater neurodevelopmental complexity. Residual differences in phenotypic complexity and prior genetic testing performed outside the practice may also have contributed to this apparently contrasting pattern.

An important counterpoint is that seizure history was associated with lower adjusted odds of undergoing genetic testing in the full cohort. This apparently paradoxical result reinforces the distinction between determinants of testing and correlates of findings among those tested. This particular cohort dataset cannot determine whether historical practice patterns, seizure-focused care, prior genetic testing performed outside the practice, insurance, family preference, chronology, or unmeasured differences in phenotypic complexity explain this association.

### 4.4. Female Sex Association

Female sex was not significantly associated with abnormal findings in the crude comparison (*p* = 0.101) but was independently associated in the primary adjusted model and remained significant in the MRI-inclusive sensitivity analysis. The direction is compatible with reports of higher molecular diagnostic yield among females in some neurodevelopmental genetics cohorts [22]. The change between crude and adjusted estimates suggests that the association is influenced by the clinical covariate structure rather than by a simple unadjusted sex difference. Because intellectual disability, dysmorphism, regression, and other phenotypic measures were unavailable, the mechanism cannot be resolved and the finding should be validated in phenotypically richer cohorts. Accordingly, residual confounding by unmeasured phenotypic severity or syndromic features remains a plausible explanation for the adjusted association with female sex.

### 4.5. EEG, MRI, and Model Discrimination

EEG abnormality was associated with abnormal genetic findings before adjustment but did not remain independently associated in the primary model, and MRI abnormality was not independently associated in the complete-case sensitivity model. These results do not imply that the EEG or MRI phenotype is irrelevant to neurogenetic diagnosis. Both variables collapse heterogeneous abnormalities into binary categories, and the dataset lacks several phenotypic features that commonly enrich genetic evaluation, including intellectual disability, dysmorphism, developmental regression, neurological examination findings, head circumference, family history, and the detailed seizure phenotype [16,18,21,22].

The AUC values of 0.590 and 0.608 for the genetic-outcome models indicate limited discrimination. These models are therefore best used to describe associations within this cohort rather than to decide which individual patients should undergo genetic testing or to predict a specific genetic diagnosis. This binary categorization may have reduced sensitivity for detecting associations confined to particular EEG or MRI phenotypes.

### 4.6. Genetic Testing Patterns and External Validity

Genetic testing was performed in 53.7% of the parent cohort and was strongly associated with having undergone EEG and MRI. This pattern is best described as the clustering of investigations within the clinical work-up, although the dataset does not retain the chronology or indication for each test. The tested subgroup should therefore not be assumed to be a random sample of all patients with ASD, and the 36.7% abnormal-finding rate should not be interpreted as the prevalence of genetic abnormalities in the entire ASD population.

External validity is also limited by the single specialty-practice setting. A pediatric neurology population may contain greater neurological complexity than community pediatric, developmental-behavioural clinics, primary-care populations, or population-based cohorts. In addition, genetic testing performed outside the practice may not have been captured uniformly. Neither source of selection can be quantified from the present dataset.

### 4.7. Clinical Implications

Current professional recommendations support etiological genetic evaluation in ASD and related neurodevelopmental disorders [11,12,13,14,15]. The present results do not support restricting testing to a narrow clinical subgroup; the primary models had limited discrimination and important phenotype variables were unavailable. Instead, the findings identify seizure history and female sex as signals that merit further study and show the breadth of abnormalities encountered in a neurology-based ASD population. The complete catalogue also demonstrates the range of genetic information that may enter neurological care, from copy-number changes and gene variants to recognizable syndromic diagnoses. Given the limited discrimination of the genetic-outcome models (AUC 0.590–0.608), these findings do not support using the measured clinical variables to select which patients should receive genetic testing. Rather, they support broad guideline-based genetic evaluation, with clinical features used to contextualize findings rather than determine access to testing.

### 4.8. Strengths and Limitations

Strengths include the large real-world ASD cohort, a genetics-specific primary outcome, the clear separation of abnormal findings from the decision to perform testing, exact analysis-specific denominators, multivariable models with collinearity checks, an MRI-inclusive sensitivity analysis, and the publication of a de-identified catalogue containing every abnormal genetic finding in the cohort. The study therefore combines a population-level association analysis with transparent reporting of the underlying genetic spectrum.

Several limitations should be considered. The genetic-testing modality was not uniformly reconstructable, preventing assay-specific rates or yields. Three abnormal results lacked a narrative finding description, and individual variants were not independently reinterpreted beyond the available clinical documentation. The analytic extract did not retain calendar dates, testing indications, or test chronology, limiting temporal inference. Testing and neurodiagnostic investigations reflected routine care rather than a research protocol, creating referral and ascertainment biases. Important phenotypic variables including intellectual ability, dysmorphism, developmental regression, neurological examination findings, head circumference, family history, ancestry, and a detailed seizure phenotype were unavailable. Finally, the cohort arose from a single specialty practice and included 170 patients aged ≥18 years, which may limit generalizability.

### 4.9. Future Research

Larger multicenter datasets could determine whether combinations of the seizure phenotype, developmental features, dysmorphism, EEG, MRI, and family history identify clinically meaningful neurogenetic subgroups while also modelling which patients enter the testing pathway.

## 5. Conclusions

In this 1884-patient ASD cohort, genetic testing was performed in 53.7% of patients and 36.7% of those tested had an abnormal genetic finding. Seizure history and female sex were independently associated with abnormal findings in the primary adjusted model and remained significant in the MRI-inclusive sensitivity analysis, whereas EEG and MRI abnormalities were not independent correlates after adjustment. The complete catalogue of all 371 abnormal findings demonstrates substantial genetic heterogeneity across copy-number, sequence, homozygosity, Fragile X-related, chromosomal, and syndromic findings. Genetic testing also clustered strongly with other neurological investigations, reflecting observed patterns of clinical work-up within this cohort rather than temporal or causal relationships. Together, these findings provide a broad real-world view of the genetic landscape encountered in an ASD neurology cohort.

## Figures and Tables

**Table 1:** Cohort characteristics by genetic-testing status.

Characteristic	Overall (N = 1884)	Genetic Testing Performed (n = 1011)	Not Genetically Tested (n = 873)	*p*
Age, years, mean (SD)	10.64 (4.81)	10.34 (4.69)	10.99 (4.92)	0.004
Male, n (%)	1424 (75.6)	752 (74.4)	672 (77.0)	0.191
Female, n (%)	460 (24.4)	259 (25.6)	201 (23.0)	
Seizure history, n (%)	339 (18.0)	172 (17.0)	167 (19.1)	0.233
EEG: normal/abnormal/not tested	1276/285/323	731/170/110	545/115/213	<0.001
MRI: normal/abnormal/not tested	382/322/1180	266/198/547	116/124/633	<0.001

Age was compared with Welch’s *t* test. Categorical *p* values are Pearson chi-square tests. EEG = electroencephalography; MRI = magnetic resonance imaging; SD = standard deviation.

**Table 2:** Spectrum of documented abnormal genetic findings.

Finding Category	Denominator	n	%	Note
Finding description available	371	368	99.2	3 records lacked narrative detail
Gene or sequence variant described	368	127	34.5	Overlapping category
Deletion described	368	77	20.9	Overlapping category
Duplication described	368	77	20.9	Overlapping category
Homozygosity/ROH described	368	61	16.6	Overlapping category
Named syndrome or condition documented	371	65	17.5	See Supplementary Table S1 for full catalogue
Fragile X-related annotation	371	27	7.3	May coexist with another abnormal finding

Categories are descriptive and not mutually exclusive. Percentages use the 368 records with an available finding description unless otherwise indicated. ROH = region of homozygosity.

**Table 3:** Characteristics of patients with normal versus abnormal genetic results.

Characteristic	Normal Result	Abnormal Result	Effect/Test	*p*
N	640	371		
Age, years, mean (SD)	10.19 (4.76)	10.61 (4.58)	Welch t = −1.40	0.161
Female, n (%)	153 (23.9)	106 (28.6)	Pearson χ^2^ = 2.68	0.101
Seizure history, n (%)	90 (14.1)	82 (22.1)	OR 1.73 (1.24–2.42)	0.001
Abnormal EEG, n/N (%)	91/570 (16.0)	79/331 (23.9)	OR 1.65 (1.18–2.31)	0.003
Abnormal MRI, n/N (%)	110/279 (39.4)	88/185 (47.6)	OR 1.39 (0.958–2.03)	0.083

EEG and MRI denominators differ because these comparisons are restricted to patients who underwent the corresponding test. Odds ratios compare seizure history vs. none, abnormal EEG vs. normal EEG, and abnormal MRI vs. normal MRI.

**Table 4:** Multivariable associations with abnormal genetic findings.

Predictor	Primary Model N = 901 aOR (95% CI), *p*	MRI-Inclusive Sensitivity N = 422 aOR (95% CI), *p*
Age, per year	1.017 (0.987–1.048), 0.270	1.013 (0.967–1.062), 0.583
Female vs. male	1.483 (1.089–2.018), 0.012	1.646 (1.039–2.609), 0.034
Seizure history	1.634 (1.136–2.352), 0.008	1.654 (1.041–2.626), 0.033
Abnormal EEG	1.373 (0.958–1.969), 0.084	1.417 (0.902–2.224), 0.130
Abnormal MRI		1.235 (0.827–1.845), 0.302
Overall model	χ^2^ = 22.2, df = 4, *p* < 0.001	χ^2^ = 16.7, df = 5, *p* = 0.005
AUC	0.590	0.608

Outcome: abnormal versus normal genetic result. Reference categories: male sex, no seizure history, normal EEG, and normal MRI. The MRI-inclusive model is a complete-case sensitivity analysis. AUC values describe in-sample discrimination. aOR = adjusted odds ratio; CI = confidence interval.

**Table 5:** Factors associated with genetic testing in the full cohort (N = 1884).

Predictor	aOR	95% CI	*p*
Age, per year	0.976	0.956–0.995	0.014
Female vs. male	1.120	0.899–1.394	0.312
Seizure history	0.683	0.531–0.879	0.003
EEG performed	2.424	1.872–3.139	<0.001
MRI performed	2.245	1.835–2.746	<0.001

Outcome: genetic testing performed versus not performed. Overall likelihood-ratio χ^2^ = 134, df = 5, *p* < 0.001; McFadden R^2^ = 0.0516; Nagelkerke R^2^ = 0.0919; AUC = 0.649.

## Data Availability

The patient-level dataset is not publicly available because of patient privacy and institutional restrictions. De-identified aggregate data may be available from the corresponding author upon reasonable request and subject to institutional and legal approval.

## Supplementary Materials

The following supporting information can be downloaded at: https://www.mdpi.com/article/10.3390/neurolint18090172/s1, Supplementary Table S1: All Recorded Genetic Findings. The spreadsheet contains all 371 abnormal genetic findings using study-specific supplement record numbers, available finding descriptions, broad descriptive categories, named syndrome or condition labels, and Fragile X annotations. Original patient identifiers and demographic linkages are omitted. The supplement is descriptive and preserves the findings as documented without independent reinterpretation.
